# An Evolutionary Study in Glyphosate Oxidoreductase Gox Highlights Distinct Orthologous Groups and Novel Conserved Motifs That Can Classify Gox and Elucidate Its Biological Role

**DOI:** 10.3390/jox15050138

**Published:** 2025-08-29

**Authors:** Marina Giannakara, Vassiliki Lila Koumandou, Louis Papageorgiou

**Affiliations:** 1 Genetics Laboratory, Department of Biotechnology, Agricultural University of Athens, Iera Odos 75, 11855 Athens, Greece; 2 Department of Biomedical Sciences, University of West Attica, Agioy Spyridonos 28, 12243 Egaleo, Greece

**Keywords:** glyphosate, evolution, sequence analysis, phylogenetics, conserved motifs

## Abstract

Glyphosate Oxidoreductase (Gox) is an enzyme known to degrade glyphosate, an intensively used wide-spectrum herbicide. Although it was first reported back in 1995, much remains unknown about its role in bacteria, its distribution across the bacterial kingdom, and its structure. This information would be valuable for better understanding the degradation pathway of glyphosate and for discovering new enzymes with the same potential. In the present study, a holistic evolutionary analysis has been performed towards identifying homologue proteins within the FAD-dependent/binding oxidoreductases family and extracting critical characteristics related to conserved protein domains and motifs that play a key role in this enzyme’s function. A total of 2220 representative protein sequences from 843 species and 10 classes of bacteria were analyzed, from which 4 protein domains, 2 characteristic/functional regions, and 8 conserved motifs were identified based on multiple sequence alignment and the annotated information from biological databases. The major goal of this study is the presentation of a novel phylogenetic tree for the Gox-related proteins to identify the major protein clusters and correlate them based on their sequence, structural, and functional information towards identifying new possible pharmacological targets that are related to this specific enzyme function. Considering the lack of information about Gox, the aim of this paper is to fill in these knowledge gaps, which can help determine the biological role of Gox and consequently better understand its function.

## 1. Introduction

Intensified agricultural activity worldwide is leading to increasing concerns about the persistence of herbicides in the environment and their negative impact on ecosystems and health [1]. Glyphosate-based herbicides are one of the most widely used pesticides across the globe, both for commercial and domestic use [2]. Glyphosate is the active ingredient in many broad-spectrum herbicides. It is a synthetic amino acid analogue of glycine bound to a phosphonic acid, and it is registered as toxic to aquatic life [3]. Despite being classified as safe by some agencies, several studies are raising awareness about its potential negative effects on crop growth, insects, and human cells [2,3,4,5,6,7,8,9,10].

Glyphosate is classified by the International Agency for Research on Cancer (IARC) as probably carcinogenic to humans, whereas the European Chemicals Agency (ECHA) does not acknowledge any carcinogenic potential. As a rather controversial compound, glyphosate has been studied for its carcinogenicity in many studies and in different concentrations, either in its pure form or included in commercial herbicide formulations. Findings suggest that the surfactants used in herbicide formulations are mainly responsible for the carcinogenic and toxic effects [11], or they may enhance such adverse effects of glyphosate [12]. However, evidence shows that glyphosate itself can also negatively affect DNA methylation in human peripheral blood mononuclear cells (PBMCs) even at low concentrations (0.5 μΜ) [8]. It has been proven to infiltrate the brain tissue in mice and hence lead to increased levels of pro-inflammatory cytokine TNFα, which is linked to neurodegenerative disorders [13]. Glyphosate can also act as an endocrine disruptor to estrogen receptors in prostate cells, causing cell death [14], and in human endometrial Ishikawa cells, acting as an aggravating factor in endometrial cancer [15].

Research on pesticide degradation, including the microorganisms, pathways, and proteins involved, can lead to significant progress in the remediation of polluted sites. So far, three bacterial enzymes are known to degrade glyphosate: C-P lyase, Glycine Oxidase (GO), and Glyphosate oxidoreductase (Gox). The first two proteins are well-characterized with known protein structures. There have also been attempts to optimize the structure of GO from *Bacillus cereus* towards more efficient glyphosate degradation [16]. However, the information about Gox protein is very limited [17]. Although AMPA (alpha-amino-3-hydroxy-5-methyl-4-isoxazolepropionic acid) and glycolate are the main products of glyphosate degradation for both GO and Gox, the catalytic reaction is different. GO produces hydrogen peroxide as a byproduct, while Gox does not. In addition, stoichiometrical differences have also been observed, as GO uses 1 mol of oxygen per 1 mol of glyphosate, whereas Gox uses 0.5 O_2_ per 1 mol of glyphosate [18].

**Figure 1:**
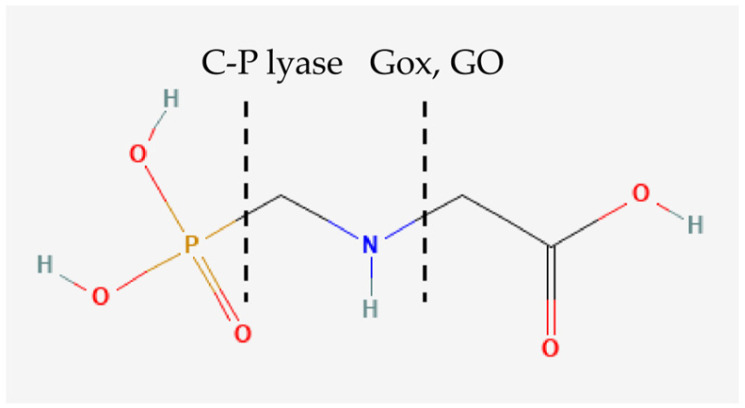
Degradation of glyphosate by the enzymes C-P lyase, Gox, and GO. Intermittent lines indicate the bonds cleaved by each enzyme. Glyphosate PubChem CID: 3496.

Gox was first reported in 1995 as part of a US patent by Monsanto, in which the Gox gene of *Ochrobactrum anthropi* LBAA, an *α-proteobacterium* of the Brucellaceae family, was used to generate glyphosate-tolerant transgenic plants. The researchers characterized Gox as an FAD-dependent protein that utilizes iminodiacetic acid (IDA) and glyphosate as substrates [19]. Degradation by Gox breaks the C-N bond of glyphosate, producing glyoxylate and AMPA (Figure 1) [19]. There is only a small number of annotated Gox proteins [17]. No experimental structure is available, although there has been a computed structural model [20]. The normal biological function of Gox in bacteria has also not been resolved yet, and the key information based on the bibliographical evidence is its function as an FAD-dependent oxidoreductase. This protein family is present in all organisms, and its members are classified with the E.C. number 1 [21], which includes a large group of proteins that utilize FAD as a cofactor and catalyze a large number of different substrates [22]. Analyzing Gox proteins and homologue sequences may provide insight into its potential biological function and unveil conserved motifs important in the structure and function of the protein.

**Figure 2:**
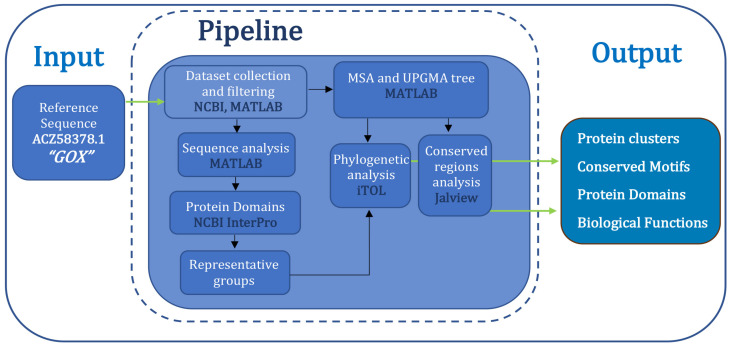
Flowchart of the Methodology followed in the present study.

To address the objectives of this study, we implemented a bioinformatics workflow including sequence retrieval, alignment, phylogenetic tree construction, and functional annotation. A summary of the workflow of our research is seen in Figure 2. This framework enabled the collection of Gox-related proteins, their classification into groups, and the identification of conserved motifs and known domains. This information can lead to potential divergence of biological roles across protein groups, providing a foundation for subsequent analyses.

## 2. Materials and Methods

### 2.1. Dataset Collection and Filtering

The Gox protein still remains insufficiently characterized, and no specific protein group has yet been identified that can adequately define its role in bacteria. Hence, we conducted a BLASTp search aiming to retrieve sequences of high similarity with Gox. The amino acid sequence of Gox ACZ58378.1 (reference sequence) from *Ochrobactrum* sp. G-1, with a length of 430 amino acids, was used as query to collect relevant bacterial sequences in the NCBI database (https://www.ncbi.nlm.nih.gov/, accessed on 28 November 2024), with more than 35% similarity. A stricter threshold than the standard 30% was applied to ensure significant homology, as all sequences belong to the same kingdom, and to ensure functional similarity at the same time [23,24,25]. Data were automatically parsed to exclude partial, synthetic, and hypothetical sequences and non-bacterial or unrelated data by employing regular expression techniques and the header information in MATLAB R2022b. The protein sequences were filtered so as to include a sequence length from 420 to 460 amino acids and then were aligned with the reference sequence and filtered based on a cutoff of less than 97% similarity. The final output was a dataset of 2200 protein sequences.

Python scripts using Python 3.12.1 with regular expressions were used to extract information from the FASTA headers regarding the protein ontologies. The taxonomy of the organisms was retrieved from the NCBI Taxonomy Database (https://www.ncbi.nlm.nih.gov/taxonomy, accessed on 25 January 2025) [26]. The retrieved ontologies that attributed the same activity to the sequences were grouped together, retaining the annotation of the most widespread term. Terms including “FAD-binding oxidoreductase”, “oxidoreductase_ FAD-binding”, and “oxidoreductase_ FAD-binding protein” were grouped under the term “FAD-binding oxidoreductase”. Detailed information about all the original ontologies is presented in Table S1.

### 2.2. Multiple Sequence Alignment, Protein Domains, and Conserved Motifs Exploration

Multiple sequence alignment (MSA) was performed using the MATLAB Bioinformatics Toolbox [27] based on a guide tree and the progressive MSA method, as in previous studies [25,28]. The results were visualized in the Jalview platform [29], and the Alignment Annotation section was used to recognize conserved motifs. The selected residues of the consensus sequence for motif inference had a Jalview score ≥9 and a frequency ≥80%. A score of 11 indicates absolute conservation, whereas a score of 10 reflects that, despite variation, physicochemical properties are conserved [29].

In order to integrate available information about the FAD-dependent proteins (known domains), 120 representative sequences were selected, including the reference sequence, so that all different ontologies and sequences from all clusters across the phylogenetic tree were represented. Domain and functional region information was retrieved for these 120 sequences from NCBI using the option “Identify conserved domains” and from InterPro (InterPro scan version: 5.68-100.0) [30,31].

### 2.3. Phylogenetic Analysis

The phylogenetic trees were constructed in the MATLAB Bioinformatics Toolbox based on the UPGMA method (Unweighted Pair-Group Method). The visualization and editing of the constructed phylogenetic tree were performed with iTOL. The iTOL annotation editor for spreadsheets was also used to divide the tree into clusters and annotate the bacterial class on the leaves [32]. The topology of the phylogenetic tree was studied to define the clusters of the monophyletic branches. The resulting clusters were associated with characteristics such as the bacterial species, protein domains, and conserved motifs.

## 3. Results

### 3.1. The Selected FAD-Dependent Proteins

**Figure 3:**
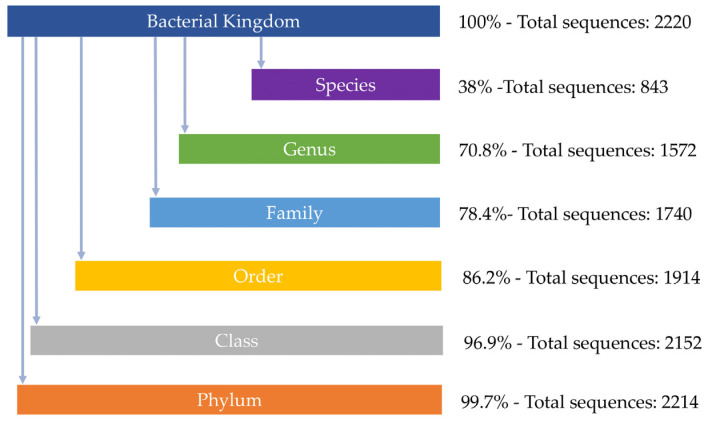
The taxonomic classification of the protein sequences within the dataset.

The NCBI protein search based on the representative ontology revealed 17 different ontologies. Following the filtering steps, the final dataset consisted of 2220 representative sequences, with a mean length of 420 amino acids (S. Data A, S. Data B). The taxonomy annotation revealed uneven levels of taxonomic information, including species, genera, families, orders, classes, and domains. 53% of the sequences did not contain any information about the bacterial species. More specifically, 0.3% of the sequences were characterized up to domain level, 2.8% to phylum level, 10.7% to class, 7.8% to order, 7.6% to family, 32.8% to genus, and 38% up to species level (Figure 3). In order to deal with this issue, we applied the nomenclature analysis, and all sequences of the dataset were evaluated based on phylum level in order to make use of the largest amount of available taxonomic information. In several instances, multiple sequences belong to the same species, such as *Rhizobium leguminosarum* with 15 sequences, *Variovorax paradoxus* with 10 sequences, *Variovorax boronicumulans* with 8 sequences, and *Agrobacterium tumefaciens* with 6 sequences (Table S2). None of these species have been reported to degrade glyphosate in the bibliography. 

Nevertheless, multiple sequences from the same species were observed. The species *Rhizobium leguminosarum* was the most frequent, with 15 sequences. It is a symbiotic nitrogen-fixing bacterium [33] and belongs to the same order as the reference sequence (Hyphomicrobiales), but in the Rhizobiaceae family. The second most frequent organism was *Variovorax paradoxus* with 10 sequences, a member of *β-proteobacteria*. It is known for its metabolic diversity and the degradation of pollutants [34]. Neither of the species has been reported to degrade glyphosate in the bibliography so far.

The most predominant taxonomic unit was unclassified *α-proteobacteria* (149 sequences), followed by the unclassified *Rhodospirillales* (68 sequences) and *Pseudomonadota* (56 sequences). As a result, 97% of the sequences are adequately characterized on the class level. The dataset consists of 10 different bacterial classes (Figure 3, Table 1). The majority of the sequences belong to α-proteobacteria (1644 sequences), followed by β- (293 sequences), γ- (195 sequences), and finally, δ-proteobacteria (9 sequences). Additional lineages include actinobacteria (3 sequences), *deinococcota* (1 sequence), *acidobacteriota* (3 sequences), *chloroflexota* (1 sequence), and *planctomycetota* (2 sequences) (Table 1 and Table S2). All are Gram-negative bacteria, with the exception of three sequences belonging to actinobacteria, which are classified as Gram-positive.

Ontology analysis can give insights about the protein type of the reference sequence and consequently contribute to the categorization of the Gox proteins based on their biological role (Table 2). However, the majority of the sequences belong to “FAD-dependent” or “FAD-binding” oxidoreductase, which is a broad group of proteins that utilize FAD as an electron donor [35]. Vague or incomplete characterizations were also included in a small number of proteins, such as “dependent oxidoreductase”, “dependent oxidoreductase family protein”, and “dehydrogenase”. The analysis revealed that “amino acid dehydrogenases”, represented by 40 sequences, was the second most predominant ontology, followed by “D-amino acid dehydrogenases” with 28 sequences. All ontologies referring to (D-) amino acid dehydrogenase activity constituted only 3.7% of the dataset. One sequence annotated to degrade glyphosate has also been included (Glyphosate oxidase ADD71136.1). Unique ontologies were also observed, appearing only once in the dataset. These include “amino acid oxidase” PZU91571, “dadA1” MDB5362004, “ketopantoate reductase PanE/ApbA family protein” AOF93253, “pyridine nucleotide-disulfide oxidoreductase family protein” AMO96042, “cytochrome C4” ESY78188, and “Glycine/D-amino acid oxidase (deaminating)” AKO97158 (Table S3). These unique ontologies were verified using each NCBI accession number as a query sequence in BLASTp against the nr database, and checking the first 5000 results. The BLASTp searches yielded “FAD -dependent”, “-binding” oxidoreductases and amino acid dehydrogenases at a percentage of 94–95%. The ontology of the query sequence was found only in 3 searches, including “amino acid oxidase” with 11 sequences of the same ontology (0.2%), “cytochrome C4” with 20 sequences (4%), and “Glycine/D-amino acid oxidase (deaminating)” with 9 sequences (0.1%). Taking into consideration the evaluation of the ontologies of the dataset, it was concluded that 11 subtypes are present in the dataset (Table 2).

### 3.2. Multiple Sequence Alignment

The MSA analysis revealed a significantly large gap in the N-terminal part of the alignment with a length of approximately 80 residues and a large gap of 56 residues towards the C-terminal end (S. Data C). This lack of conversation possibly indicates loops or unstructured regions in the proteins. Highly conserved regions are located after the N-terminal gap and before the C-terminal gap in the alignment, suggesting the existence of the catalytic site or structurally important regions. Less or not conserved regions are also observed in between. The distribution of hydrophobic and hydrophilic residues is almost even across the MSA, with the exception of the N-terminal end of the alignment, which consists of mostly hydrophobic amino acids. Moreover, all the protein sequences have been aligned with the reference Gox protein towards estimating the overall protein similarity for each bacterial class (Table 1, S. Data D).

### 3.3. Phylogenetic Analysis

**Figure 4:**
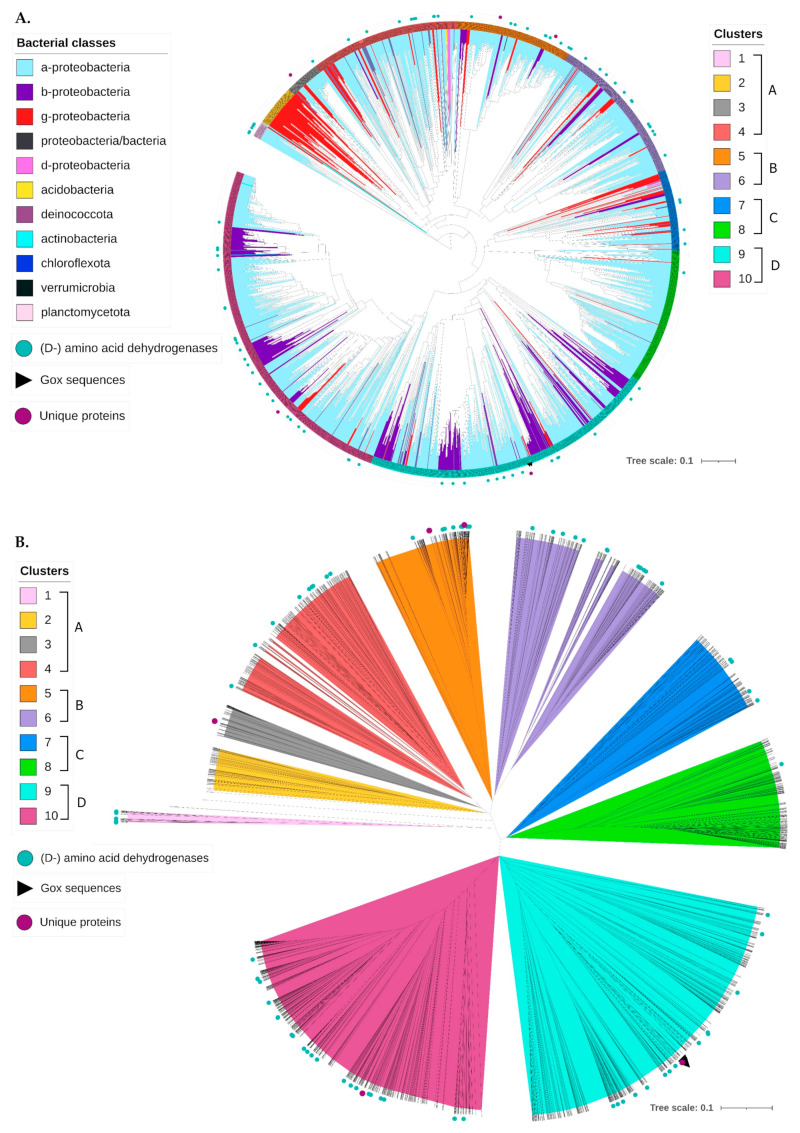
(**A**) The phylogenetic tree based on the 2220 homologue representative sequences of the dataset. The clusters are represented by the different colors of the periphery. Each branch is colored according to the bacterial group of each leaf. The sequences of amino acid dehydrogenases, Gox, and those with unique annotations are demonstrated with shape labels. (**B**) The unrooted phylogenetic tree. Four groups are formed: Clusters 1–4 (group A), Clusters 5 and 6 (group B), Clusters 7 and 8 (group C), and Clusters 9 and 10 (group D). The reference sequence and glyphosate oxidase from ADD71136.1 are placed in Cluster 9. The phylogenetic trees are also available in higher resolution as separate files in the Supplementary Materials.

The resulting phylogenetic tree (available in Newick format in S. Data E) consists of distinct clusters, i.e., groups of clades clearly separated from each other (Figure 4A and Figure S1). 10 clusters were defined in order to investigate whether the members of each cluster share common domains or taxonomy. The unrooted tree (Figure 4B and Figure S2) reveals 4 major cluster groups: (A) Clusters 1, 2, 3, and 4; (B) Clusters 5 and 6; (C) Clusters 7 and 8; (D) Clusters 9 and 10. Cluster 1 appears as the most ancient in the tree, followed by the FAD-binding oxidoreductase of an unknown actinobacterium (MDA2980030.1) and Clusters 2 and 3. The analysis revealed that the different bacterial classes are not clustered separately, as each cluster contains sequences of more than one class. The groups with the least number of sequences (such as chloroflexota) do not form distinct branches but are dispersed into different clusters. Therefore, although the taxonomy of each sequence affects its placement on the phylogenetic tree, it is not the only factor, as it does not lead to a clear separation of each bacterial class. The D-amino acid dehydrogenases and amino acid dehydrogenases, collectively named as (D-) amino acid dehydrogenases, also do not form one separate cluster. No cluster is observed that exclusively has (D-) amino acid dehydrogenases (Table S4). Complete absence of (D-) amino acid dehydrogenases is observed in Clusters 2 and 3, whereas Cluster 8 includes only one sequence. For the rest of the Clusters, the presence ranges from 3% in Cluster 7 to 22% in Cluster 1. The median is 3.6%, taking into consideration all 10 Clusters. The percentage in Clusters 4 and 10 is 4%, and for Clusters 5 and 6, it is 5%. Therefore, (D-) amino acid dehydrogenases may share some common elements with the other members of the same cluster, which differentiate them from the rest of the sequences. The reference sequence and Glyphosate oxidase ADD71136.1 are located in Cluster 9 among other sequences of α-proteobacteria, but sequences of β- and γ-proteobacteria are also present in the same cluster. DadA1 is also present in Cluster 9.

### 3.4. Conserved Motifs: Multiple Alignment Results

**Figure 5:**
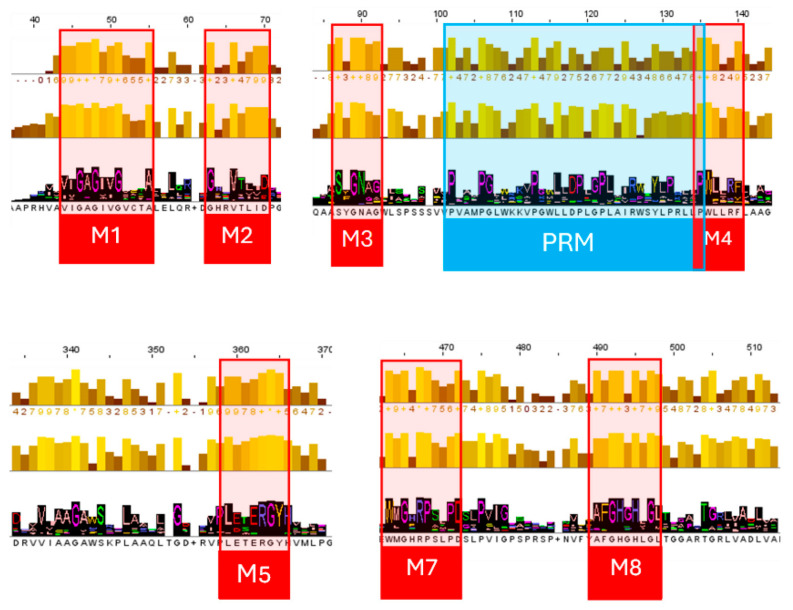
Highly conserved motifs were identified on the consensus sequence based on the MSA of the 2220 protein sequences. The red boxes indicate the placement of the motifs on the alignment (M1–M8) and the Pro-rich motif (PRM). Conserved residues were extracted based on the Jalview conservation score and frequency.

The consensus protein sequence revealed 8 conserved motifs. Based on their order of appearance in the alignment, they were named M1 to M8 (Figure 5).

Most of the identified highly conserved motifs are placed towards the N-terminal end of the alignment. The motif [V/I]-[I/V]-G-A-G-x-[V/I]-G-x-x-x-A (M1) matches the known [V/I]-x-G-x1–2-G-x-x-G-x-x-x-[G/A] motif, an extended version of G-x_1-2_-G-x-x-G and G-x-x-x-A. These motifs are associated with the FAD-binding region of proteins and indicate the presence of a Rossman fold [36]. In our alignmnet, the second variable residue of the extended motif and the fourth one are conserved (I/V and A, respectively). Interestingly, the motif often coincided with a predicted signal peptide and, more specifically, with the H-region of the predicted signal peptides.

Following the initial conserved region, two additional motifs were identified: G-x-x-V-x-x-[I/V]-[D/E] (M2) 12 residues downstream of M1 and S-x-G-N-x-[G/A] (M3) 15 residues downstream of M2. While these motifs are highly conserved across the aligned sequences, they do not correspond to any previously reported functional motifs in the literature. In the next 40 residues a cluster of 7 Proline residues was also identified. Three of them were highly conserved (Jalview score 10, with a minimum frequency of 96.76%), whereas the other three Pro residues appeared in 87.03% (P119,) 96.53% (P122), and 80.6% (P131) of the sequences. These findings suggest a possible proline-rich motif (PRM), consisting of two P-x-x-x-P and one P-x-x-P motif, combined into a Proline-rich conserved region P-x-x-x-P-(x)6-P-(x)5-P-x-x-P-(x)8-P-x-x-x-P. Sequences lacking Pro at a specific position do not cluster together in the phylogenetic tree, except for those missing Pro119 in the alignment, which group within Cluster 2. The presence of the PRM may hint at protein-protein interactions, as PRM is frequently reported to play a crucial role in such binding sites [37] or to further molecular recognition interactions, including interactions between bacteria and their host [38].

A fourth motif was identified overlapping with the last Pro of the RPM, P-W-x-x-x-[F/Y] (M4). Although the full motif is not described in any research, a shorter motif, W-x-x-x-[F/Y], is well studied in peroxisomal proteins in mammals and yeast, as its presence is essential in protein-protein interactions [39]. Nevertheless, no information is available about its presence in bacteria.

In the central segment of the alignment, two additional motifs, L-[E/D]-x-x-R-G-Y (M5) and [L/I]-R-x-x-G-x-x-E-x-[A/G] (M6), were also found to be highly conserved. However, no information is available in the literature regarding their presence in other proteins or their role. Additionally, the motifs W-[M/L]-G-x-R-P-x-x-x-D (M7) and A-x-G-H-x-H-x-G-L (M8) appear conserved towards the C-terminal part of the sequences, which were also not identified in the literature.

### 3.5. Protein Domains

Only one domain was retrieved from NCBI, which spans most of the length of the sequences, named DadA (Glycine/D-amino acid oxidase (deaminating), COG0665). It is frequently separated into 2 parts by a gap. Our initial results in NCBI additionally included the DAO domain (FAD-dependent oxidoreductase family, pfam01266). However, the same sequences are not associated with the DAO domain anymore, as it has been replaced by the DadA domain, possibly due to recent changes in the database (Table S5). Nevertheless, DAO still remains an active entry in the NCBI database, suggesting that this change was an adjustment for a more precise annotation.

Additional information was retrieved with InterPro. In this database, the DAO domain is integrated as “FAD-dep_OxRdtase” (IPR006076), spanning most of the length of the sequences. In addition, all sequences are characterized by the domain SSF54373 of the SUPERFAMILY database (FAD-linked reductases, C-terminal domain), which is placed in the C-terminal end and overlaps with FAD-dep_OxRdtase. It has an approximate length of 85–88 amino acids, and it is considered part of the alpha and beta (a+b) proteins class.

Information predicting the localization of the protein in the cell was also identified. Cytoplasmic (intracellular) and non-cytoplasmic domains (outside of the cytoplasm) were identified, as well as signaling peptides and transmembrane or membrane-bound regions. The length of the cytoplasmic domains varies from 6 to 391 amino acids, whereas non-cytoplasmic domains range from 5 to 415 amino acids. The presence of the non-cytoplasmic domain indicates that this part of the protein is extracellular. TMhelix is also the only predicted region in the reference sequence, with a length of 18 amino acids. The region at the N-terminal end of the sequences is often characterized by the domains SIGNAL-PEPTIDE and/or SignalP_noTM. They both refer to signal peptides (SPs), and they are identified by the databases PHOBIUS and SIGNALP EUK [40], respectively. SPs are important for the correct translocation of newly synthesized proteins to the cell membrane. Once the translocation of the protein is completed, the signal peptide is cleaved by signal peptidases [41]. The presence of the signal peptide in these sequences suggests that these proteins are possibly located in the cell membrane or excreted. This is useful especially for the sequences for which no other domain is predicted (cytoplasmic, non-cytoplasmic domain, or transmembrane regions), as it could reveal the localization of the protein in the cell. The length of SIGNAL-PEPTIDE and SignalP_noTM domains varies from 16 to 29 and 16 to 27 amino acids, respectively. For simplicity reasons, characteristic regions of the same type deriving from different databases were labelled with the same annotation. Therefore, SIGNAL_PEPTIDE and SignalP_noTM were merged into one domain (Signal Peptide), and the TMhelix domain and TRANSMEMBRANE are also represented by one domain (Transmembrane).

**Figure 6:**
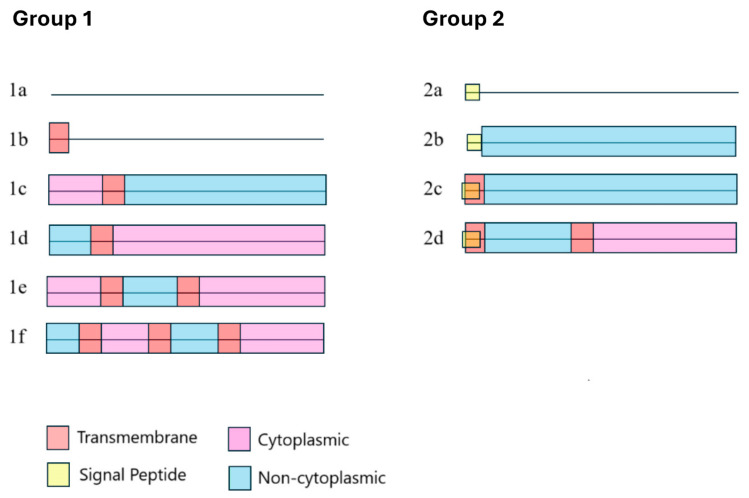
The three major domains and regions/sites (occurring in more than 2 sequences) that have been identified in the studied dataset based on InterPro and the different combinations found within the representative protein sequences. The horizontal dark lines represent the length of the sequences, and the colored boxes represent the domains. The position and length of the domains are approximate. The reference sequence belongs to group 1b. Group 1 includes all sequences that lack the signal peptide, and Group 2 includes all sequences with it.

The sequences are divided into 2 groups depending on the presence or absence of the signal peptide (Figure 6). Further classification was done based on the presence of the cytoplasmic or non-cytoplasmic domain, resulting in 6 subcategories in group 1 and 4 subcategories in group 2. This categorization of the sequences combined with their placement on the tree reveals an uneven distribution of the predicted domains across the tree (Table 3). It shows a gradual transition from simple domain combinations in Clusters 1–5 (groups 1a, 1b, and 2b) to more complicated ones in Clusters 6–10 (groups 1c-1e and 2c and 2d) (Table 4). Therefore, the simplest forms may also be more ancient, as they appear in the clusters closer to the root of the tree, whereas the more complicated ones appear later. Additionally, the cytoplasmic domain and the transmembrane regions are absent in clusters 1 and 2. The combination of both non-cytoplasmic and cytoplasmic regions seems to be related to the evolution of the bacterial phyla and is more evident in group 1, which lacks the signal peptide. Group 1a, which represents the absence of any domain or other predicted region, is present in most bacterial phyla. In contrast, groups 1c-1f are found in α-, β-, and γ-proteobacteria, which evolved more recently. Nevertheless, the presence of extra transmembrane regions and cytoplasmic/non-cytoplasmic domains is not associated with extra amino acids in these sequences, compared to the sequences with simpler domain architecture.

The proteins of unique annotated ontologies do not differ from the rest of the sequences in terms of the retrieved domains, as they belong to groups 1a and 2b. A search in the InterPro database revealed that sequences of “ketopantoate reductase”, “PanE/ApbA family protein”, “pyridine nucleotide-disulfide oxidoreductase”, and “protein cytochrome C4” contain domains that are attributed to their annotated protein type. These domains were not found in the sequences of our dataset in the Interpro Scan results, suggesting a possible annotation issue of these sequences.

Most sequences are characterized by the signature “D-Amino Acid Oxidase, subunit A, domain 2” (CATH Superfamily 3.30.9.10) in the CATH-Gene database [42]. It is placed after the first 150 residues of the sequences and ends towards the C-terminus. A gap of usually 75–77 amino acids interrupts the signature, dividing it into two parts. The CATH superfamily 3.30.9.10 is associated with 376 different CATH structural domains. That is, a model domain appears in the InterPro results for each CATH-Gene signature. In our dataset, the signature derives from chains of different structures and protein annotations, including a bacterial “L-proline dehydrogenase”, “Glycine oxidase”, “Sarcosine oxidase”, “heterotetrameric sarcosine oxidase”, and a mitochondrial “dimethylglycine dehydrogenase” from rat. They are all associated with the “Alpha Beta 2-Layer Sandwich structure”.

The signature PTHR13847 (sarcosine dehydrogenase-related) was detected in 59 of the 60 sequences, spanning almost the entire sequence length. The site profile PS512579 (PROKAR_LIPOPROTEIN), associated with prokaryotic membrane lipoproteins, is located near the N-terminal region. Additionally, the conserved domain PS51257, present at the N-terminus of FAD-dependent oxidoreductases, consists of 21–27 amino acids and represents a predicted cleavage site for signal peptidase II in prokaryotic membrane proteins [43]. Searches against PDB showed that there are currently no PDB structures of these proteins or of any closely related ones.

## 4. Discussion

In the present study, we report a first-time attempt to categorize Gox from the bacterium *Ochrobactrum* sp. G-1 into a specific group of proteins and characterize it. Despite its specific name, there is no information about the biological role of Gox or which protein group it belongs to. Homologous sequences were retrieved and filtered to a dataset that constitutes a novel protein group related to Gox. This includes a variety of bacterial classes, but mostly representatives of α-proteobacteria. Although most of the sequences of the dataset were of unspecified function and annotated with the term “FAD dependent/binding oxidoreductases”, our analysis included (D-) amino acid dehydrogenases and an amino acid oxidase. Further individual instances of specific protein types were also observed, which were possibly the result of mis-annotation or an extremely low similarity with other proteins of the same type. Additionally, although GO is also known to degrade glyphosate [44], no GO sequences were found in the BLASTp representatives, suggesting that Gox does not share any close similarity with GO. This is also validated by the fact that Gox and GO act on the C-N bond of glyphosate with different mechanisms [16].

Gox was predicted by InterPro to contain a transmembrane part with a helical structure in the N-terminus. Additional transmembrane regions and/or SPs are found in the sequences of the dataset, whereas non-cytoplasmic domains indicate extracellular components. These findings suggest that the sequences collected can either have transmembrane parts, be excreted, or be embedded in the membrane. The prediction of a non-cytoplasmic domain across almost all the length of the protein raises the question of whether these proteins are the extracellular component of a larger protein complex. Two different groups were defined based on the presence and combination of the known domains, which show an increase in the complexity of the domains from the most ancient clusters to the most recent ones.

The conserved motifs observed in the multiple alignment also added new information about the sequences of the dataset. Although most of the conserved motifs are not reported in the literature, the possible presence of the Rossman fold was inferred by the motif [V/I]-x-G-x1–2-G-x-x-G-x-x-x-[G/A]. The Rossman fold is linked to the FAD-binding region of proteins, and it is considered an ancient structural fold [45]. The potential of protein-binding properties is suggested by a possible proline-rich motif (PRM). In addition, a well-characterized peroxisomal targeting signal motif has also been identified, which is not known to be functionally present in bacteria yet. New and yet unknown motifs have also been suggested, which should be further studied as to their contribution to the function of the active site of the protein or as a means to identify this group of proteins.

The phylogenetic tree did not lead to defined protein groups in terms of taxonomy, protein annotation, or known domains. The sequences of the same bacterial groups formed smaller clusters distributed across the tree, whereas (D-) dehydrogenases were distributed across the tree unevenly, suggesting a potential differentiating trait that separates these (D-) dehydrogenases and groups them together with the other members of the cluster.

Further analysis is essential, as it may elucidate the structural and functional characteristics of Gox and its homologue sequences. This may lead to the characterization of proteins of unidentified role or to a new group of proteins that is involved in glyphosate degradation. Such knowledge can contribute to the study of the pathways involved in glyphosate degradation and detoxification, the fate of the pesticide in the environment, and the development of bioremediation programs for glyphosate-contaminated regions. In order to achieve this aim, the next steps include precise protein annotation, the definition of clear characteristics of the proteins, a complete characterization of the pathway involved, as well as the utilization of omics-based techniques. The two known motifs and the experimentally known structures of the homologous sequences can help enhance the precision of the two available computed 3D structures, which are based on homology modelling [20] and AI by AlphaFold [46]. As a result, a more reliable 3D protein model can be generated and consequently better predict the active site of the protein.

### Limitations of the Present Study

The results of the present in silico study provide key characteristics of the Gox protein, its homologue selected group, and its representative domains and conserved motifs. This can provide a solid foundation toward developing practical applications in the field, such as enzyme biosensors for glyphosate detection [47] or biocatalysts for its degradation [48]. However, such conclusions are drawn via a computational approach based on the existing information in the databases. Therefore, biochemical and functional validations via in vivo experiments are essential. A complete characterization of Gox would include determining protein localization with reporter fusion, such as the utilization of green fluorescent protein (GFP) [49], as well as protein expression and purification in order to determine the crystallographic structure of Gox. The evolutionary and phylogenetic conclusions are based on the highest resolved taxonomic level for the majority of the sequences (class) due to the different levels of taxonomic annotation of the homologous sequences. Only 38% of the sequences are characterized at the species level, but it is expected that more sequences will be characterized at the species level in the future, allowing more detailed phylogenetic conclusions.

## 5. Conclusions

Taken together, there is a notable lack of information about the functionality of Gox in bacteria. Our research shows that even the homologue sequences of Gox have an unknown role in bacteria. An attempt to profile these sequences was performed by identifying the known conserved domains in databases and inferring the conserved motifs within all proteins of the closest representatives for Gox based on their multiple alignment.

The presence of amino acid dehydrogenases further indicates a possible connection with Gox, but additional analysis is needed to confirm this. The present work represents a first step toward understanding the biological role of Gox and defining a group of bacterial sequences with potential new glyphosate-degrading candidates. It provides basic evidence for a future, more detailed functional characterization of Gox and its homologue sequences to better understand their role and to investigate the possible functional pathways in bacteria. Our findings, along with the proposed novel group of homologues, provide a solid foundation toward developing practical applications in the field, such as enzyme biosensors for glyphosate detection or biocatalysts for its degradation.

## Figures and Tables

**Table 1:** The distribution of the sequences and ontologies of the dataset per bacterial class.

Phyla	Total Seq.	Classes	Total Seq.	Homology Percent	Ontologies
Pseudomonadota (Proteobacteriota)	2199	a-proteobacteria ^1^	1644	36–84%	D-amino acid dehydrogenase
					D-amino acid dehydrogenase small subunit
					D-amino acid dehydrogenase 1
					FAD-binding/dependent oxidoreductase
					* Glycine/D-amino acid oxidase (deaminating)
					* Gox (Glyphosate oxidoreductase)
					amino acid dehydrogenase
					amino acid oxidase
					* cytochrome C4
					* dadA1
					* ketopantoate reductase PanE/ApbA family protein
		b-proteobacteria ^1^	293	37–56%	D-amino acid dehydrogenase
					D-amino acid dehydrogenase 1
					FAD-binding/dependent oxidoreductase
					amino acid dehydrogenase
					* pyridine nucleotide-disulfide oxidoreductase family protein
		g-proteobacteria ^1^	195	37–69%	FAD-binding/dependent oxidoreductase
					amino acid dehydrogenase
		d-proteobacteria ^1^	9	41–46%	FAD-binding/dependent oxidoreductase
		Undefined Proteobacteriota ^1^	58	36–48%	FAD-binding/dependent oxidoreductase
-	-	Undefined Bacteria ^4^	10	40–41%	D-amino acid dehydrogenase small subunit
					FAD-binding/dependent oxidoreductase
					* Gox (FAD-dependent glyphosate oxidase)
Actinomycetota	3	Actinobacteria ^2^	3	39–45%	FAD-binding/dependent oxidoreductase
					amino acid dehydrogenase
Acidobacteriota ^1^	3	-	3	40–43%	FAD-binding/dependent oxidoreductase
Planctomycetota	2	-	2	41–43%	FAD-binding/dependent oxidoreductase
Chloroflexota ^3^	1	-	1	40%	amino acid dehydrogenase
Verrucomicrobiota	1	Verrucomicrobia ^1^	1	41%	amino acid dehydrogenase
Deinococcota ^1^	1	-	1	40%	FAD-dependent oxidoreductase

^1^ Gram-negative. ^2^ Gram positive. ^3^ Monoderms. ^4^ Not defined. * Only 1 annotated protein type present in the dataset.

**Table 2:** The different ontologies of the dataset; ^a^ protein types grouped as “(D-) amino acid dehydrogenase”; ^b^ protein types grouped in one ontology; ^c^ protein types grouped as “Gox”.

Protein Type	Count
FAD-dependent/binding oxidoreductase	2121
amino acid dehydrogenase ^a^	40
D-amino acid dehydrogenase ^a^	28
D-amino acid dehydrogenase 1 ^a^	8
dependent oxidoreductase ^b^	4
dependent oxidoreductase family protein ^b^	3
D-amino acid dehydrogenase small subunit ^a^	3
putative D-amino acid dehydrogenase protein ^a^	3
Dehydrogenase	2
amino acid oxidase	1
cytochrome C4	1
dadA1	1
FAD-dependent glyphosate oxidase ^c^	1
Glycine/D-amino acid oxidase _deaminating_	1
Gox ^c^	1
ketopantoate reductase PanE/ApbA family protein	1
pyridine nucleotide-disulfide oxidoreductase family protein	1

**Table 3:** The distribution of the different domains across the 10 clusters of the phylogenetic tree, which were retrieved via InterPro (Figure 6). Clusters A–D represent the groups as they appear in the phylogenetic tree in Figure 4.

		Clusters									
		A				B		C		D	
		1	2	3	4	5	6	7	8	9	10
Group 1	1a	x	x	x	x	x	x	x	x	x	x
	1b		x		x		x	x	x		x
	1c			x		x		x		x	
	1d			x		x		x		x	
	1e								x	x	
	1f							x			
Group 2	2a										
	2b	x	x		x	x	x	x		x	x
	2c				x	x			x	x	x
	2d						x				

**Table 4:** The distribution of the different groups of Figure 6 across the different bacterial phyla. The order of appearance of the phyla reflects their evolutionary order.

		Group 1						Group			
Phyla		1a	1b	1c	1d	1e	1f	2a	2b	2c	2d
	g-proteobacteria	x	x	x			x		x		x
	b-proteobacteria	x	x					x	x	x	
	a-proteobacteria	x	x	x	x	x		x	x	x	
	d-proteobacteria	x									
	acidobacteriota	x									
	deinococcota									x	
	chloroflexota	x									
	actinobacteria (actinomycetota)	x									
	planctomycetota									x	
	verrucomicrobiota								x		

## Data Availability

The original contributions presented in this study are included in the article and in the Supplementary Dataset. Further inquiries can be directed to the corresponding author(s).

## Supplementary Materials

The following supporting information can be downloaded at: https://www.mdpi.com/article/10.3390/jox15050138/s1; Figure S1; High resolution image of the phylogenetic tree of Figure 4A; Figure S2: High resolution image of the phylogenetic tree of Figure 4B; Table S1: The different ontologies retrieved from the dataset. Duplicate annotations are included, which were either differentiated by the presence of a dash (-) or with similar terms, such as FAD dependent oxidoreductase and FAD-binding oxidoreductase. These instances were considered as one ontology in our study; Table S2: The different taxonomic units present in the dataset; Table S3: The unique ontologies of the dataset. The results using the corresponding accession numbers for BLASTp searches against the nr database are presented; the searches did not return any proteins annotated with the protein ontology given in the 5 out of 6 representatives; Table S4: The percentage of (D-) amino acid dehydrogenases on each cluster of the phylogenetic tree; Table S5: Examples of the sequences, for which the domains have changed in NCBI, as the DAO domain was replaced with a DadA domain; S. Data A: The final dataset used in the analysis; S. Data B: the final dataset with full header information, including the labels used in the analysis, the NCBI accession number and protein ontology; S. Data C: The MSA of all protein sequences in the dataset; S. Data D: The similarity percentage of each sequence of the dataset with the reference Gox protein sequence; S. Data E: The UPGMA tree of the homologue protein sequences with Gox protein.

## Abbreviations

The following abbreviations are used in this manuscript:

AMPA	alpha-amino-3-hydroxy-5-methyl-4-isoxazolepropionic acid
ECHA	European Chemicals Agency
GO	Glycine Oxidase
Gox	Glyphosate oxidoreductase
IARC	International Agency for Research on Cancer
IDA	iminodiacetic acid
MSA	Multiple sequence alignment
PBMCs	peripheral blood mononuclear cells
PRM	proline-rich motif
