# Supplementary material for: An Evolutionary Study in Glyphosate Oxidoreductase Gox Highlights Distinct Orthologous Groups and Novel Conserved Motifs That Can Classify Gox and Elucidate Its Biological Role
Source: J Xenobiot. 2025 Aug 29;15(5):138. doi: 10.3390/jox15050138 (PMC12452685; doi:10.3390/jox15050138)
Supplement: Supplementary file 1 [file jox-15-00138-s001.zip › Table_S1.pdf]

Table S1. The different ontologies retrieved the final dataset. Duplicate annotations are included, which were either differentiated by the presence of a dash (-) or with similar terms, such as FAD dependent oxidoreductase and FAD-binding oxidoreductase. These instances were considered as one ontology in our study.

Protein Type	Count
amino acid dehydrogenase	28
amino acid dehydrogenase	12
amino acid oxidase	1
cytochrome C4	1
dadA1	1
D-amino acid dehydrogenase	2
D-amino acid dehydrogenase	1
D-amino acid dehydrogenase	24
D-amino acid dehydrogenase	1
D-amino acid dehydrogenase 1	7
D-amino acid dehydrogenase 1	1
D-amino acid dehydrogenase small subunit	3
dehydrogenase	2
dependent oxidoreductase	4
dependent oxidoreductase family protein	3
FAD dependent oxidoreductase	4
FAD-binding oxidoreductase	994
FAD-binding oxidoreductase	42
FAD-binding oxidoreductase	20
FAD-dependent glyphosate oxidase	1
FAD-dependent oxidoreductase	991
FAD-dependent oxidoreductase	38
FAD-dependent oxidoreductase	29
Glycine/D-amino acid oxidase _deaminating_	1
Gox	1
ketopantoate reductase PanE/ApbA family protein	1
NADP/FAD-dependent oxidoreductase	1
oxidoreductase_ FAD-binding	1
oxidoreductase_ FAD-binding protein	1
putative D-amino acid dehydrogenase protein	3
pyridine nucleotide-disulfide oxidoreductase family protein	1
