# Supplementary material for: An Evolutionary Study in Glyphosate Oxidoreductase Gox Highlights Distinct Orthologous Groups and Novel Conserved Motifs That Can Classify Gox and Elucidate Its Biological Role
Source: J Xenobiot. 2025 Aug 29;15(5):138. doi: 10.3390/jox15050138 (PMC12452685; doi:10.3390/jox15050138)
Supplement: Supplementary file 1 [file jox-15-00138-s001.zip › Table_S3.pdf]

Table S3. The unique ontologies of the final dataset. The results using the corresponding accession numbers for BLASTp searches against the nr database are presented; the searches did not return any proteins annotated with the protein ontology given in the 5 out of 6 representatives.

**Protein ontology**	**NCBI Accession number**	**Same annotation in the BLASTp results**
cytochrome C4	ESY78188	No
ketopantoate reductase PanE/ApbA family member	AOF93253	No
pyridine nucleotide disulfide oxidoreductase family member	AMO96042	No
Glycine/D-amino_acid oxidase (deaminating)	AKO97158	No
dadA1	MDB5362004	No
amino acid oxidase	PZU91571	Yes
