# Supplementary material for: An Evolutionary Study in Glyphosate Oxidoreductase Gox Highlights Distinct Orthologous Groups and Novel Conserved Motifs That Can Classify Gox and Elucidate Its Biological Role
Source: J Xenobiot. 2025 Aug 29;15(5):138. doi: 10.3390/jox15050138 (PMC12452685; doi:10.3390/jox15050138)
Supplement: Supplementary file 1 [file jox-15-00138-s001.zip › Table_S4.pdf]

Table S4. The percentage of (D-) amino acid dehydrogenases on each cluster of the phylogenetic tree.

**Cluster**	**Number of sequences**	**Cluster leaves**	**Percentage**
1	6	27	22.22%
2	0	66	0.00%
3	0	57	0.00%
4	10	241	4.15%
5	10	187	5.35%
6	13	244	5.33%
7	4	131	3.05%
8	1	220	0.45%
9	14	480	2.92%
10	24	567	4.23%
**Sum**	82	2220	3.69%
