# Supplementary material for: An Evolutionary Study in Glyphosate Oxidoreductase Gox Highlights Distinct Orthologous Groups and Novel Conserved Motifs That Can Classify Gox and Elucidate Its Biological Role
Source: J Xenobiot. 2025 Aug 29;15(5):138. doi: 10.3390/jox15050138 (PMC12452685; doi:10.3390/jox15050138)
Supplement: Supplementary file 1 [file jox-15-00138-s001.zip › Table_S5.pdf]

Table S5. Examples of the sequences, for which the domains have changed in NCBI, as the DAO domain was replaced with a DadA domain.

**Protein Type**	**Accession Number (NCBI)**	**Length**	**Domains-NCBI**	**NCBI domains updated: Accessed 19-06-2024**
FAD_binding_ oxidoreductase	WP_164006702.1	430	DAO (15-430)	DadA: 15-429
amino_acid_dehydrogenase	HCL67388.1	413	DAO (6-413)	DadA 6-412
D_amino_acid_dehydrogenase	BBK36167.1	420	DAO (14-420)	DadA 14-419
dadA1*	MDB5362004.1	415	DAO (3-415)	DadA 1-414
cytochrome_C4*	ESY78188.1	411	DAO (6-393)	DadA 3-410
NAD_P_/FAD_dependent_oxidoreductase	WP_126110285.1	410	NAD(P)/FAD-dependent oxidoreductase (1-410), DAO (2-392), NAD_binding_8 (5-91)	DadA: 1-46- GAP (47-95)- DadA: 96-409 NAD_binding_8 (5-91)
Glycine/D_amino_acid_oxidase__deaminating*	AKO97158.1	425	DAO (16-425)	DadA 18-424
D_amino_acid_dehydrogenase_1	PPR76660.1	448	DAO (40-448)	DadA 37-447
ketopantoate_reductase_PanE/ApbA_family_protein	AOF93253.1	412	DAO (4-412)	DadA 4-57 GAP(58-99) DadA 100-411
