# Supplementary figures and images for: An Evolutionary Study in Glyphosate Oxidoreductase Gox Highlights Distinct Orthologous Groups and Novel Conserved Motifs That Can Classify Gox and Elucidate Its Biological Role
Source: J Xenobiot. 2025 Aug 29;15(5):138. doi: 10.3390/jox15050138 (PMC12452685; doi:10.3390/jox15050138)

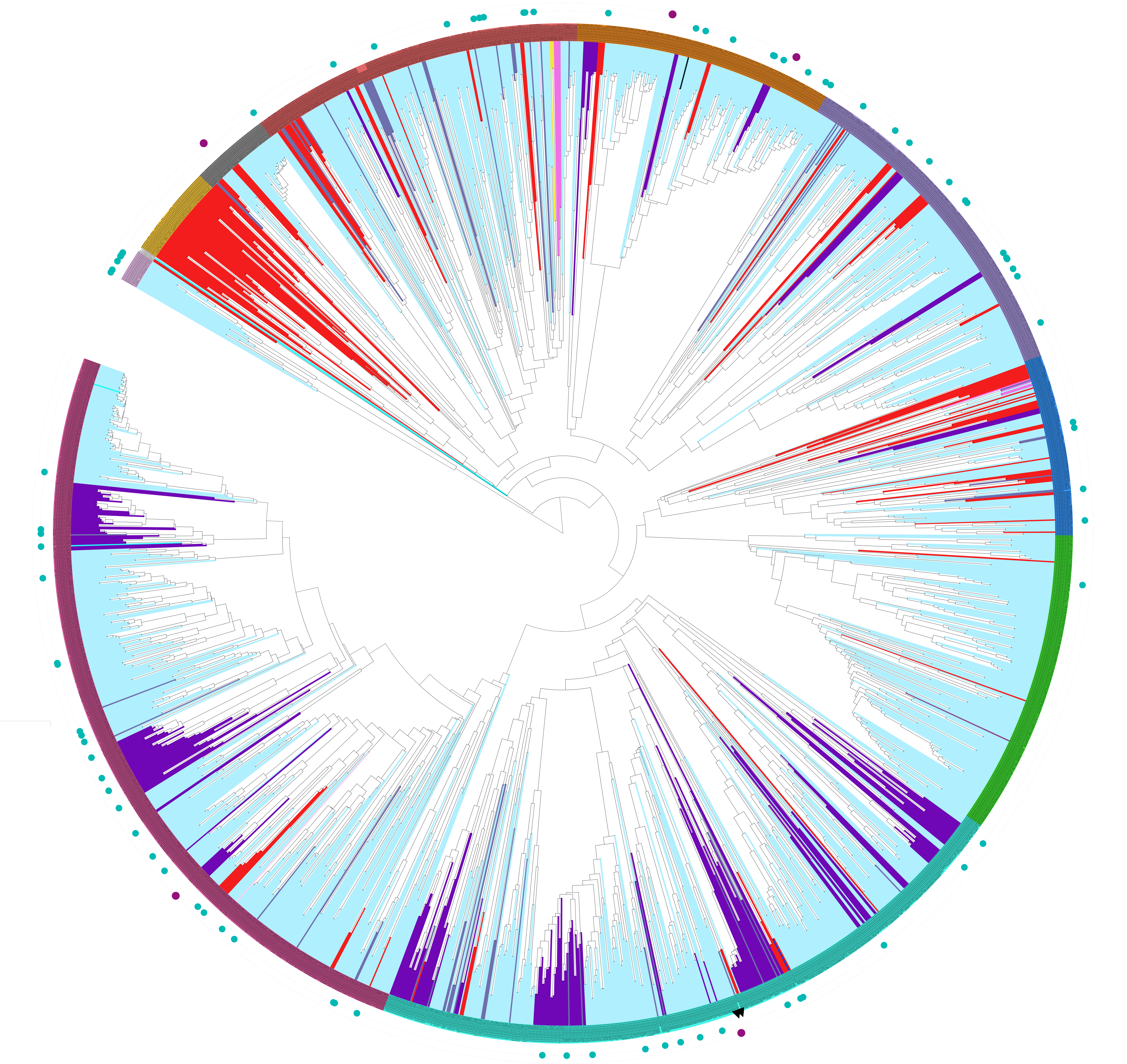

Supplement: Supplementary file 1 [file jox-15-00138-s001.zip › Figure_S1_RV.jpg]
